# Immunoregulatory effects of multipotent adult progenitor cells in a porcine ex vivo lung perfusion model

**DOI:** 10.1186/s13287-017-0603-5

**Published:** 2017-07-05

**Authors:** An Martens, Sofie Ordies, Bart M. Vanaudenaerde, Stijn E. Verleden, Robin Vos, Dirk E. Van Raemdonck, Geert M. Verleden, Valerie D. Roobrouck, Sandra Claes, Dominique Schols, Eric Verbeken, Catherine M. Verfaillie, Arne P. Neyrinck

**Affiliations:** 1 Laboratory of Anesthesiology and Algology, Department of Cardiovascular Sciences, Katholieke Universiteit Leuven and University Hospitals Leuven, Herestraat 49, 3000 Leuven, Belgium; 2 Leuven Lung Transplant Unit, Katholieke Universiteit Leuven, Leuven, Belgium; 3 Laboratory of Pneumology, Department of Clinical and Experimental Medicine, Lung Transplant Unit, Katholieke Universiteit Leuven and University Hospitals Leuven, Leuven, Belgium; 4 Laboratory of Experimental Thoracic Surgery, Department of Clinical and Experimental Medicine, Katholieke Universiteit Leuven and University Hospitals Leuven, Leuven, Belgium; 5 ReGenesys BVBA, Leuven, Belgium; 6 Laboratory of Virology and Chemotherapy (Rega Institute), Department of Microbiology and Immunology, Katholieke Universiteit Leuven, Leuven, Belgium; 7 Department of Histopathology, University Hospitals Leuven, Leuven, Belgium; 8 Stem Cell Institute Leuven, Department of Development and Regeneration, KU Leuven-University of Leuven, Leuven, Belgium

**Keywords:** Multipotent adult progenitor cell, Ex vivo lung perfusion, Immunomodulation, Ischemia-reperfusion injury, Porcine, Immunoregulation

## Abstract

**Background:**

Primary graft dysfunction (PGD) is considered to be the end result of an inflammatory response targeting the new lung allograft after transplant. Previous research has indicated that MAPC cell therapy might attenuate this injury by its paracrine effects on the pro-/anti-inflammatory balance. This study aims to investigate the immunoregulatory capacities of MAPC cells in PGD when administered in the airways.

**Methods:**

Lungs of domestic pigs (n = 6/group) were subjected to 90 minutes of warm ischemia. Lungs were cold flushed, cannulated on ice and placed on EVLP for 6 hours. At the start of EVLP, 40 ml of an albumin-plasmalyte mixture was distributed in the airways (CONTR group). In the MAPC cell group, 150 million MAPC cells (ReGenesys/Athersys, Cleveland, OH, USA) were added to this mixture. At the end of EVLP, a physiological evaluation (pulmonary vascular resistance, lung compliance, PaO_2_/FiO_2_), wet-to-dry weight ratio (W/D) sampling and a multiplex analysis of bronchoalveolar lavage (BAL) (2 × 30 ml) was performed.

**Results:**

Pulmonary vascular resistance, lung compliance, PaO_2_/FiO_2_ and W/D were not statistically different at the end of EVLP between both groups. BAL neutrophilia was significantly reduced in the MAPC cell group. Moreover, there was a significant decrease in TNF-α, IL-1β and IFN-γ in the BAL, but not in IFN-α; whereas IL-4, IL-10 and IL-8 were below the detection limit.

**Conclusions:**

Although no physiologic effect of MAPC cell distribution in the airways was detected during EVLP, we observed a reduction in pro-inflammatory cytokines and neutrophils in BAL in the MAPC cell group. This effect on the innate immune system might play an important role in critically modifying the process of PGD after transplantation. Further experiments will have to elucidate the immunoregulatory effect of MAPC cell administration on graft function after transplantation.

## Background

In the year 2016, lung transplantation has grown into a successful treatment option for patients with end-stage pulmonary disease. However, severe primary graft dysfunction (PGD) still occurs in up to 30% of transplanted patients. And although there is a low mortality of PGD due to successful supportive therapy nowadays, patients with severe PGD have poorer long-term outcome and a higher risk for developing chronic lung allograft dysfunction (CLAD). PGD is the end result of ischemia-reperfusion injury (IRI) attacking the integrity of the capillary-alveolar membrane leading to pulmonary edema and impaired oxygenation [1, 2]. It is based on an inflammatory cascade that is triggered by hypoxic stress and activation of donor macrophages, which attracts many recipient neutrophils to the donor lung upon reperfusion in the recipient chest. Bone marrow-derived mesenchymal cells possess immunoregulatory capabilities and could therefore be of particular interest to attenuate PGD. They are already found to be a successful treatment option for patients suffering from acute respiratory distress syndrome, which shares a similar inflammatory pathophysiology with PGD [3–5]. More specifically, pilot studies show that they can reduce IRI inherent to solid organ transplantation in the lung [6–8], but also in other organ systems [9, 10]. Here, their beneficial effects result from paracrine mechanisms and cell-cell interaction rather than engraftment and repair of diseased tissue [11]. An altered inflammatory balance, with a decrease in pro-inflammatory and increase in anti-inflammatory cytokines, is observed [6]. In hypoxic conditions, such as ischemic injury models, secretion of growth factors such as VEGF and ANG-1 can stimulate angiogenesis and tissue repair [8, 12]. Mordant et al. recently published their results on mesenchymal stem cell (MSC) administration to a porcine donor lung on ex vivo lung perfusion (EVLP), which resulted in a reduction of interleukin (IL)-8, however, there was no effect on physiological parameters detected [13]. The role in a reduction of inflammatory cytokines in an acellular ex vivo perfusion set-up therefore still has to be unraveled.

There are two types of bone marrow-derived cells that are well characterized and could be good candidates for immunoregulation of the IRI pathophysiology: the mesenchymal stem cell (MSC) and multipotent adult progenitor cell (MAPC) [14]. Both have similar immunoregulatory capabilities, but are characterized as individual cell types and adopt different phenotypes under certain culture conditions [15]. One of the most advantageous characteristics of the MAPC cells is their large proliferation capacity and low senescence. Therefore, large batches of stem cells can be produced from one healthy donor [16, 17]. A clinical-grade MAPC product (MultiStem®, Athersys, Cleveland, OH, USA) has been developed for phase I and II clinical testing. The MultiStem clinical-grade product is based on MAPC cell isolation and expansion protocols under good manufacturing practice conditions [18, 19]. Our previous research indicates that intratracheal administration of cells holds the greatest potential to reduce IRI based on a reduction in lung edema formation. However, after administration of 10 million MAPC cells in the airways, a physiological improvement of lung function was not detected nor was there an alteration in the inflammatory environment of the lung tissue. Therefore, in this study we increased the dosing regimen to an intermediate dose of 3.75 million/kg in order to investigate a physiological improvement of lung function and to study this mechanism based on immunoregulation. Also for other solid organ transplant research, this intermediate dosing regimen of 3.5–4.5 million cells/kg has led to an improved graft function after transplantation [4, 9, 10].

In order to administer these cells, ex vivo lung perfusion (EVLP) has been put forward as a treatment platform of ex vivo organ reconditioning. EVLP is a preservation technique of donor lungs in normothermic conditions, allowing for continuous evaluation of the donor lung by perfusing and ventilating the graft in an ex vivo lung perfusion device. EVLP allows for assessment of higher-risk donor lungs and might serve as the ideal platform for active treatment since donor and recipient remain unharmed, and the treatment effect can be immediately evaluated by interpretation of the physiological parameters [20, 21].

The immunoregulatory properties of MAPC cells have not been widely studied in preclinical large animal models and most preliminary theories of working mechanisms are based on in vitro and rodent models. Therefore, in this large animal study, we aim to investigate if MAPC cell delivery in the airways during EVLP (i.e., post-conditioning), can modulate the inflammatory process linked to IRI by immunoregulatory effects.

## Methods

This experimental study was performed in compliance with the European Directive 2010/63/EU on the protection of animals used for scientific purposes. The principles of laboratory animal care published by the National Institute of Health Volume 25, No. 28 (revised 1996) were followed. Local ethics approval was obtained at the research institute (Animal Ethics Committee Katholieke Universiteit Leuven Belgium, NTS P043/2014).

### Donor procedure

Domestic pigs Topig 20 (mean 40.8 kg) were divided into two groups (n = 6/group). Animals were anesthetized with an intramuscular injection of 5 mg/kg Zoletil 100 (Virbac, Carros, France) and 3 mg/kg Xyl-M 2% (VMD, Arendonk, Belgium). Anaesthesia was maintained using 10 mg/kg/h propofol, 20 μg/kg/h fentanyl and intermittent boli of pancuronium 2 mg for muscle relaxation. Animals were intubated with a 7.0 mm endotracheal tube and ventilated (Aestiva 3000; GE Healthcare Europe GmbH, Little Chalfont, UK) with a tidal volume (TV) of 8 ml/kg, positive end-expiratory pressure (PEEP) of 5 cmH_2_O and FiO_2_ of 30%. Respiratory rate (RR) was adjusted to the end-tidal carbon dioxide (ETCO_2_) (45–55 mmHg). Blood pressure was monitored invasively in the right carotid artery. All animals died of cardiac arrest, which was induced by direct electrical stimulation of the myocardium with an electrical pulse generator that led to ventricular fibrillation. Animals were disconnected from the ventilator when cardiac arrest was induced. Prior to cardiac arrest, all animals were heparinized with 300 IU/kg.

Following cardiac arrest in the donor, grafts were left untouched in the deceased donor for 90 minutes after which they were flushed antegradely with 50 ml/kg cold thromethamol-buffered OCS Solution (Transmedics, Andover, MA, USA). The heart-lung block was excised and a retrograde flush (1 L thromethamol-buffered OCS solution) was performed at the back table. Lungs were instrumented on ice for a short period of time (mean 64.4 minutes) while the XVIVO (Göteborg, Sweden) cannulas were secured in the pulmonary artery and atrial cuff. An 8.0-mm ET tube was secured in the trachea. The donor procedure was performed as previously described [22].

### Multipotent adult progenitor cell preparation

Human MAPC cells were isolated by Athersys/Regenesys (Athersys, Cleveland, OH, USA; Regenesys, Heverlee, Belgium) from bone marrow of healthy volunteers. A research-grade variant of the clinical product was used: cells were cultured under the same medium conditions as MultiStem®, but not in a Good Manufacturing Practice (GMP) environment. Isolation and cultivation of the MAPC cells were based on previously published protocols [19, 23]. The Quantum Cell Expansion System (Terumo BCT, Lakewood, CO, USA) was used for ex vivo expansion of large batches of MAPC. All cell batches were subjected to several quality control assays to test if all MAPC cell criteria were met. First of all, cell quality is assessed by measuring viability and plating efficiency post-thawing. Cells were thawed at a population doubling of 29.4 and had a post-thawing viability of ≥ 96%. Second, cells are identified using qPCR and flow cytometry to test both negative and positive markers [15, 24]. A tube formation assay is done to define the proangiogenic activity [25], a CFSE assay [26] is performed for evaluating the immunoregulatory capacity.

### Ex vivo lung perfusion

**Fig. 1:**
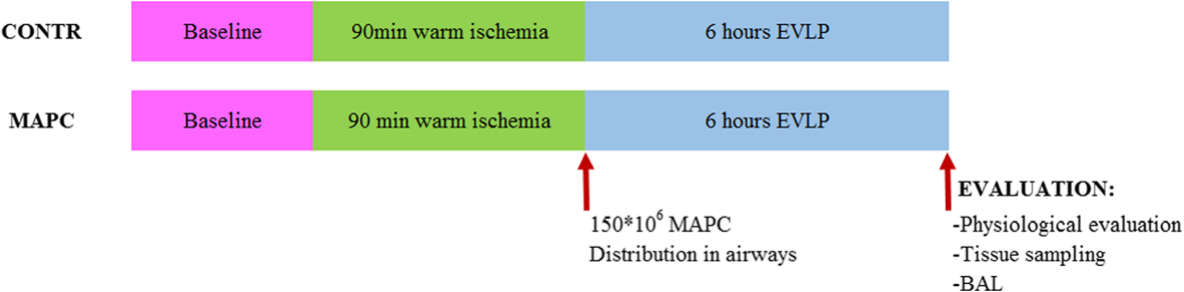
Study protocol with n = 6/group. *BAL* bronchoalveolar lavage, *CONTR* control group, *EVPL* ex vivo lung perfusion, *MAPC* multipotent adult progenitor cell

Lungs are perfused ex vivo with an acellular albumin containing dextran solution. The production of the perfusate and technique of EVLP are performed as described previously [27]. After a 1-hour rewarming period and slow increase of the flow to 40% of the estimated cardiac output (calculated as 100 ml/kg) lungs were further perfused and evaluated for 6 hours in total. Once the outflow temperature reaches 34 °C, ventilation was started with 7 ml/kg tidal volume for 7 breaths/min with 5 cmH_2_O PEEP. In the CONTR group, 40 ml of acellular albumin (2.5%)-plasmalyte mixture was distributed with a bronchoscope throughout the lung when ventilation was started. In the MAPC cell group, 150 million MAPC cells were added to this mixture. The study protocol is outlined in Fig. 1. Cells were thawed, PBS washed and diluted in 40 ml of the albumin-plasmalyte mixture after confirming that the donor lungs were free of adhesions or infiltrates. During 6 hours of EVLP, dynamic lung compliance (Compl), oxygenation [partial oxygen pressure over fractional inspired oxygen concentration (PaO_2_/FiO_2_)] and pulmonary vascular resistance (PVR) was recorded hourly.

### Tissue sampling

At the end of the experiment, tissue samples were taken for histological evaluation and wet-to-dry-weight (W/D) ratio calculation (after 48 hours in the oven at 80 °C). Pathology samples are scored by an experienced pathologist (EV) for congestion, neutrophil influx and necrosis. Bronchoalveolar lavage (BAL) with two times 30 cc saline 0.9% was performed in the right middle lobe. Pooled fractions were returned and a cytospin (100 μl) was stained with Diff-Quick (Dade Behring, Newark, NJ, USA) to perform total and differential cell counts. The BAL supernatant was analyzed with a porcine multiplex ELISA kit for IL-1β, IL-4, IL-8, IL-10, interferon (IFN)-γ, IFN-α and tumor necrosis factor alpha (TNF-α) according to the manufacturer’s protocol (Thermo Fisher Scientific Inc., Waltham, MA, USA). The left lung was inflated at 25 cmH_2_O, frozen solid in the fumes of liquid nitrogen and scanned with Siemens Somaton CT scanner (Siemens Healthcare, Erlangen, Germany). Lung mass, volume, and density were measured on the basis of the computed tomography (CT) scan, using imaging software (Horos®) in which the lung is manually delineated and the number of voxels and mean density of the voxels within the volume is determined [28].

### Statistical analysis

All data are expressed as median with interquartile range (IQR) when depicting physiological variables in time or as a scatter plot with median and IQR when comparing variables at the end of the experiment (GraphPad Prism 4, GraphPad Software Inc., La Jolla, CA, USA).

Mann-Whitney tests were conducted in GraphPad to compare data at the end of EVLP. We analyzed end-experimental parameters only to dichotomize between acceptable and non-acceptable lungs. Baseline parameters of the donor animals are described as median (25% QI – 75% QI) and are analyzed with the same statistical test. The level of statistical significance was set at *p* < 0.05.

## Results

### Baseline

Baseline animal parameters and the perfusate are similar between both groups (Table 1).

**Table 1:** Baseline animal parameters and perfusate composition

	CONTR	MAPC	*p* value
Baseline animal			
Weight (kg)	40.3 (38.8–41.2)	42.3 (40.2–43.1)	0.10
Vt (mL/kg)	7.9 (7.8–8.0)	7.9 (7.8–7.9)	0.85
HR (bpm)	88 (73–118)	107 (94–148)	0.17
MAP (mmHg)	102 (90–113)	96 (77–106)	0.42
Peak Awp (cmH_2_O)	19.5 (18.8–21.3)	18.5 (17.5–19.3)	0.16
PaO_2_/FiO_2_ (mmHg)	413 (399–455)	405 (376–421)	0.37
WBC (10^9^/L)	19.8 (14.3–24.7)	18.5 (15.2–21.2)	0.62
Neutrophils (%)	42.5 (32.3–51.0)	46.0 (37.5–52.5)	0.68
Perfusate composition			
Albumin (g/L)	65.5 (64.0–66.9)	66.8 (63.5–68.8)	0.47
Osmolality (mmol/kg H_2_O)	318 (614–320)	318 (315–319)	0.85
Na (mmol/L)	154 (154–156)	155 (153–156)	0.79
K (mmol/L)	3.5 (3.5–3.6)	3.5 (3.5–3.6)	0.51
CI (mmol/L)	105 (104–106)	105 (104–105)	0.29
Bicarbonate (mmol/L)	31.2 (29.6–31.9)	32.6 (30.6–33.1)	0.16
Ca (mmol/L)	0.57 (0.55–0.57)	0.53 (0.52–0.56)	0.10
Glucose (mg/dL)	233 (230–239)	236 (229–248)	0.98

Comparable animal weight and baseline hemodynamic parameters. No signs of systemic inflammation (normal WBC count, normal neutrophil count, normal temperature). Perfusate composition is similar between both groups for albumin, osmolality, glucose, and electrolytes.

*CONTR* control group, *MAPC* multipotent adult progenitor cell, *Vt* tidal volume, *HR* heart rate, *MAP* mean arterial pressure, *Peak AwP* peak airway pressure, *PaO*_*2*_ */Fi*O_2_ partial oxygen pressure/fractional inspired oxygen concentration, *WBC* white blood cell count, *Na* sodium, *K* potassium, *Cl* chloride, *Ca* calcium

### Physiological assessment

**Fig. 2:**
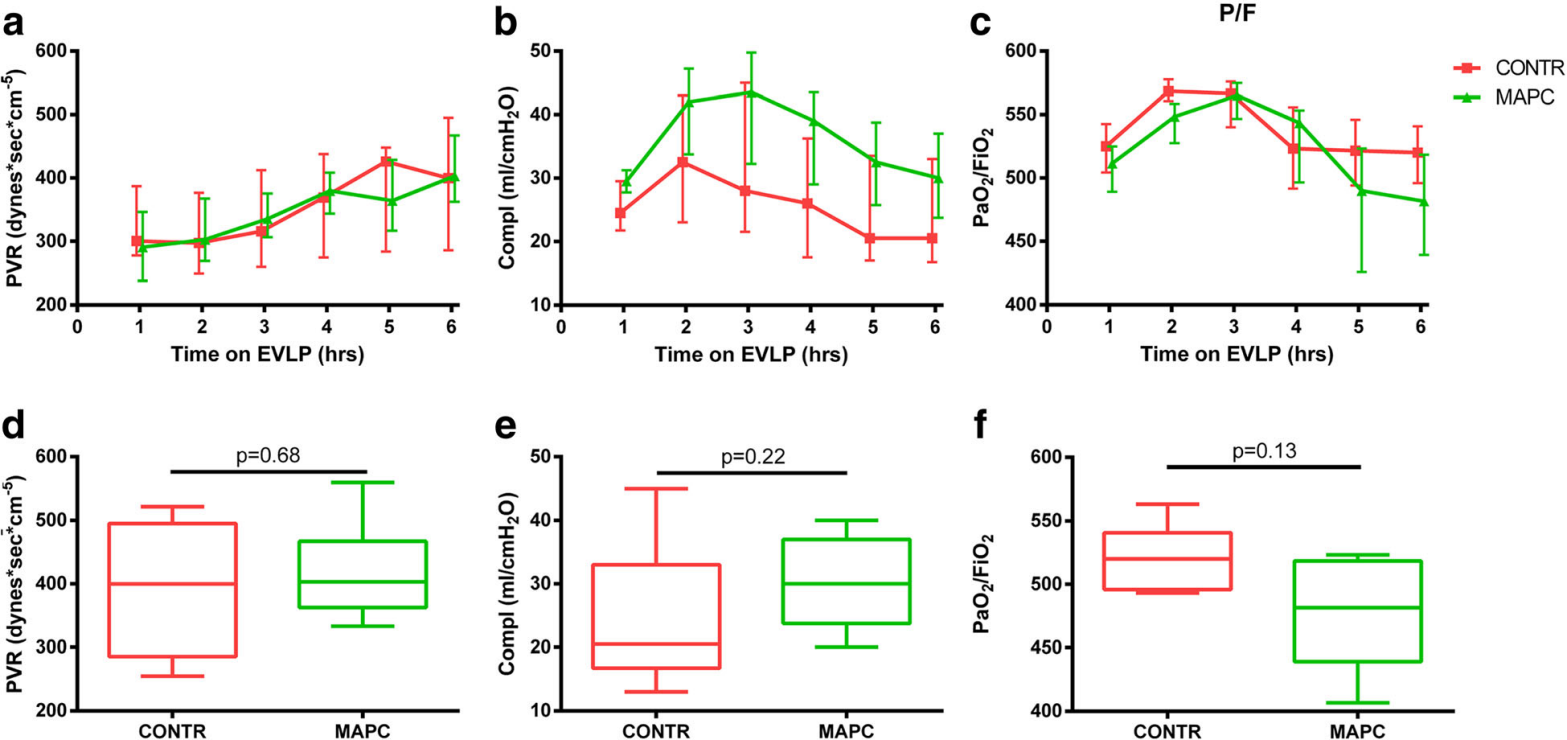
Monitoring of PVR (**a**), Compl (**b**) and PaO_2_/FiO_2_ (**c**) during 6 hours of EVLP. The final assessment at the end of EVLP did not show any statistical difference between both groups for PVR (**d**), Compl (**e**) or PaO_2_/FiO_2_ (**f**). *BAL* bronchoalveolar lavage, *Compl* dynamic lung compliance, *CONTR* control group, *EVPL* ex vivo lung perfusion, *MAPC* multipotent adult progenitor cell, *PaO*_*2*_ */FiO*_*2*_ partial oxygen pressure/fractional inspired oxygen concentration, *PVR* pulmonary vascular resistance

Physiological parameters PVR, Compl, and PaO_2_/FiO_2_ are depicted over time (hours on EVLP) in panel a-c of Fig. 2. All data points are depicted as median +/- IQR.

Physiologic parameters at the end of EVLP, are depicted in panel d-f. No statistical differences were detected in PVR (*p* = 0.68), Compl (*p* = 0.22) or PaO_2_/FiO_2_ (*p* = 0.13) between the two groups.

### Lung edema estimation

**Fig. 3:**
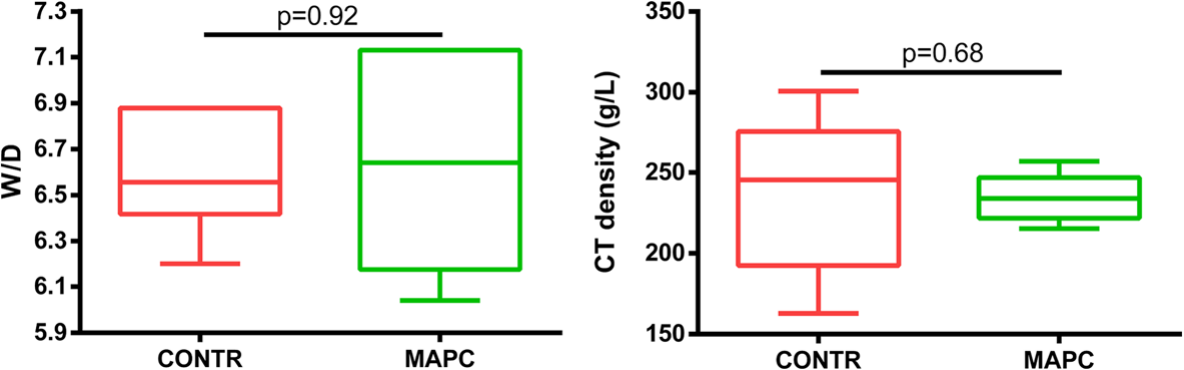
Lung edema, estimated here with W/D and CT density, is not reduced in the MAPC cell group. Data are depicted as boxplot, and compared with a Mann-Whitney test. *CONTR* control group, *CT* computed tomography, *MAPC* multipotent adult progenitor cell, *W/D* wet-to-dry weight ratio

W/D of the tissue sample of the right lower lobe and CT density calculation of the left inflated frozen lung did not reveal a statistical difference between both groups (Fig. 3). Administration of 150 million stem cells did not result in attenuation of lung edema formation in the warm ischemic-injured porcine lungs.

### Histology

**Fig. 4:**
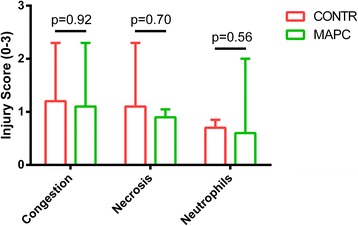
No differences were seen in the injury severity score for congestion, presence of necrosis, and neutrophils. *CONTR* control group, *MAPC* multipotent adult progenitor cell

No significant differences were detected in the injury scores for both groups for the presence of congestion (*p* = 0.92), necrotic cells (*p* = 0.70) or influx of neutrophils (*p* = 0.56) (Fig. 4).

### Inflammation bronchoalveolar lavage fluid

**Fig. 5:**
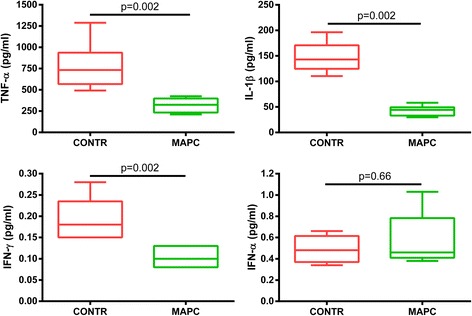
Significant reduction of inflammatory markers TNF-α, IL-1β, and IFN-γ in BAL *CONTR* control group, *IFN* interferon, *IL* interleukin, *MAPC* multipotent adult progenitor cell, *TNF-α* tumor necrosis factor alpha

Quantitative determination of IL-1β, IL-4, IL-8, IL-10, IFN-γ, IFN-α, and TNF-α in BAL fluid showed a significant reduction of TNF-α, IL-1β, and IFN-γ while IFN-α was similar in both groups (Fig. 5). IL-4, IL-8, and IL-10 were below the detection limit.

Cell pellet analysis of the returned fractions of the BAL fluid showed a trend toward reduction in total cell count in the MAPC cell group (*p* = 0.09). Also, differential cell count showed a significant reduction in neutrophils (*p* = 0.02). Results are depicted as median (25% QI – 75% QI) in Table 2.

**Table 2:** Total cell count (TCC) and differential cell count of BAL fluid cell pellet

	CONTR	MAPC	*p* value
TCC (×10^6^ cells/ml)	1.2 (0.9–1.5)	0.8 (0.7–1.0)	0.09
Macrophages (%)	88.5 (84.2–91.9)	95.3 (91.4–97.2)	0.06
Neutrophils (%)	7.7 (6.5–13.5)	1.7 (1.2–4.4)	0.02
Lymphocytes (%)	2.5 (0.8–3.9)	3.0 (1.7–3.7)	0.79

*BAL* bronchoalveolar lavage, *CONTR* control group, *MAPC* multipotent adult progenitor cell

## Discussion

In this study, we report the immunoregulatory effects on pulmonary IRI, of MAPC cell administration in the airways. Distribution of 150 × 10^6^ MAPC cells in the airways of warm-ischemic porcine donor lungs resulted in a decreased concentration of TNF-α, IL-1β, and IFN-γ in the BAL supernatant. Also, a decreased percentage of neutrophils and a trend to a lower total cell count in the BAL cell pellet was observed. The attenuation of this inflammatory response to warm-ischemic injury, was not reflected by an improvement in physiologic parameters, histology or lung edema during EVLP assessment.

Up to now, bone marrow-derived MSC are most frequently used for immunoregulation of the inflammatory response to pulmonary ischemia-reperfusion injury [29, 30]. MAPC cells are also bone marrow-derived mesenchymal cells and share many similarities with the MSC, however in different culture conditions they adapt different phenotypes [15]. The interest in the use of cellular treatment for ischemia-reperfusion injury results from in vitro evidence of their immunoregulatory capacities published in the last decade [31]. This immunoregulation is mainly described as the suppression of regulatory T cells in vitro [16] while the interaction with neutrophils, the most important effector cell in IRI, is largely unexplored. MSC and MAPC share similar immunoregulatory capabilities, although MAPC cells are found to be more potent compared to MSC in rhesus monkeys [32], human MSC and MAPC share similar immunoregulatory capabilities [23, 26, 33]. However, MAPC cell therapy is privileged over MSC for clinical translation since their low senescence and high population doubling allows for banking of large batches of cells from a single donor [15]. A clinical-grade product of MAPC, MultiStem, is produced by Athersys and is already being tested in phase I and II clinical trials (Athersys, Cleveland, OH, USA). Therefore, we chose this cell type as bone marrow-derived cellular treatment for attenuation of ischemia-reperfusion injury in lung transplantation. Also, xenotransplantation and allotransplantation of these cells is thought to be safe due to a low expression of MHC I and lack of MHC II expression [14, 33]. These immune-privileged properties avoid recognition by the recipient’s immune system, and therefore, this immune mismatch is generally believed not to be an increased risk for patients.

We used a validated injury model with exposure of the lung graft to 90 minutes of warm ischemia [22]. This resulted in an accumulation of lung edema when perfused for 6 hours on EVLP, which is reflected by a high W/D (median 6.6 in CONTR) and high CT density (median 245 g/L in CONTR). An inflammatory cascade with infiltration of neutrophils was launched upon reperfusion of the donor lung on EVLP, even though we worked with an acellular perfusate. This is shown by an increased percentage of neutrophils in the BAL in the CONTR group (median 7.7% vs. 1.7%). Most likely, these cells were trapped in the microvasculature of the lung despite adequate antegrade and retrograde flush of the lung upon procurement. Also, general inflammatory markers such as TNF-α, IFN-γ, IFN-α, and IL-1β were increased in CONTR showing activation of the innate immune system with interaction of macrophages, lymphocytes, endothelial cells, and epithelial cells. A porcine multiplex analysis was used (Thermo Fisher Scientific Inc., Waltham, MA, USA), however cross-reactivity between swine and human is very typical, therefore we did not use human-specific ELISA kits to get an insight into the inflammatory injury microenvironment of the lung.

While Mordant et al. could show an IL-8 reduction in the perfusate in their model of MSC therapy during EVLP [13], the detection limit of 0.56 pg/ml for IL-8 was not reached in our BAL sample. Therefore, we cannot speculate on the effect on IL-8 and its neutrophil chemotaxis effect. We did report a reduction in general inflammatory markers involved in the innate immune system, such as TNF-α, IL-1β, IFN-γ, and saw a significant reduction in neutrophils in the BAL sample. While these immunoregulatory effects are highly significant, they were not reflected by an improved graft function on EVLP evaluation. The exact effect of this immunoregulatory effect, therefore, has to be further unraveled in a transplant experiment where the lung graft is reperfused with a pulsatile flow of whole blood and the full impact of IRI can be studied.

In our model, MAPC cells were administered in the airways. This route of administration is based on the pathological findings of ischemia-reperfusion injury and acute respiratory distress syndrome, which share a similar pathophysiology. In the acute phase of IRI, epithelial denudation leads to loss of the epithelial cell layer over much of the basal membrane (in combination with lesser endothelial damage such as cell swelling, widening of the intercellular junctions, and increased number of pinocytotic vesicles) [34, 35]. Since diffuse alveolar damage in the acute phase is thus primarily based on epithelial cell injury, we aimed at targeting the epithelial side of the alveolar membrane with our cellular therapy.

Also, dose-response studies will have to be further conducted to determine the maximal effect. In this study, an intermediate dose of 3.75 million MAPC/kg body weight was administered. In mice studies, 0.5 to 2 million cells are usually administered, which corresponds to 100 to 200 million cells/kg. These doses are probably irrelevant for clinical practice since we also have to watch out for entrapment of these large cells (15–19 μm) in the microvasculature of the lung with microthrombi formation, increased PVR and hydrostatic pulmonary edema as potential dangerous consequence. A low dose of 0.24 million cells/kg has been shown not to be effective to tackle IRI as reported by our group previously [36]. It might therefore be that one should be looking for an optimal dose instead of a linear dose-response effect. A dose-response study would therefore be much more insightful to distinguish between low efficacy or underdosing of our investigated therapy in this experimental animal model.

La Francesca et al. previously investigated the effect of MAPC instillation in the airways to improve lung quality for transplantation [7]. However, in their study, MAPC cells were instilled in the left lower lobe while the right lower lobe was used as control. Due to perfusion differences between the left and right lung on EVLP, we often see that after prolonged EVLP the left lung is macroscopically better preserved than the right lung. Therefore, we wanted to investigate the effect of MAPC cells instilled in the airways with two different test groups rather to use the right lower lobe as a control.

Finally, we acknowledge that biodistribution of our cells could not be shown. Entrapment and localization of the administered cells will have to be investigated to understand the underlying mechanisms of the immunoregulatory capacities of cellular therapy.

## Conclusions

In conclusion, we can state that, although no physiologic effect of immunoregulation was detected during EVLP, we did observe a reduction in pro-inflammatory cytokines and neutrophils in the BAL after MAPC cell distribution in the airways indicating a regulatory effect on the innate immune system. This effect might play an important role in critically modifying the process of PGD early after transplantation [37]. The innate immune system is also involved in the etiology of bronchiolitis obliterans syndrome (BOS) [38], and therefore MAPC cell administration early in the process of lung transplantation might have an effect on the development of BOS, the main predictor of long-term outcome. Further experiments will have to elucidate the effect of MAPC cell administration on graft function after transplantation.

## Abbreviations

BAL: Bronchoalveolar lavage
BOS: Bronchiolitis obliterans syndrome
Ca: Calcium
Cl: Chloride
CLAD: Chronic lung allograft dysfunction
Compl: Dynamic lung compliance
CT: Computed tomography
ETCO_2_: End-tidal carbon dioxide
EVLP: Ex vivo lung perfusion
HR: Heart rate
IFN: Interferon
IL: Interleukin
IQR: Interquartile range
IRI: Ischemia-reperfusion injury
K: Potassium
MAP: Mean arterial pressure
MAPC: Multipotent adult progenitor cell
MSC: Mesenchymal stem cell
Na: Sodium
PaO_2_/FiO_2_: Partial oxygen pressure over fractional inspired oxygen concentration
Peak AwP: Peak airway pressure
PEEP: Positive end-expiratory pressure
PGD: Primary graft dysfunction
PVR: Pulmonary vascular resistance
RR: Respiratory rate
TCC: Total cell count
TNF-α: Tumor necrosis factor alpha
TV: Tidal volume
W/D: Wet-to-dry weight ratio
WBC: White blood cell count
